# Hydrothermal synthesis and structural characterization of ammonium ion-templated lanthanide(III) carboxylate-phosphonates

**DOI:** 10.3389/fchem.2014.00094

**Published:** 2014-11-05

**Authors:** Ayi A. Ayi, Tiffany L. Kinnibrugh, Abraham Clearfield

**Affiliations:** ^1^Department of Pure and Applied Chemistry, University of CalabarCalabar, Nigeria; ^2^Department of Chemistry, Texas A&M UniversityCollege Station, TX, USA

**Keywords:** hydrothermal synthesis, carboxylate-phosphonate hybrid material, metal organophosphonate frameworks, aminopolyacid ligand, flexible coordination

## Abstract

Using *N*-(phosphonomethyl) iminodiacetic acid (H_4_PMIDA), as a complexing agent, two new complexes, (NH_4_)La(PMIDA)(H_2_O)•H_2_O, **1** and (NH_4_)Yb(PMIDA), **2** have been synthesized hydrothermally. In both compounds, the metal ions are trapped by a three five-membered chelate rings by the chelating PMIDA anions giving a tricapped trigonal prismatic LaO_8_N and monocapped trigonal prismatic YbO_6_N geometries for **1** and **2**, respectively. The structure of **1** consists of La(PMIDA)(H_2_O) chelating units, linked together by the phosphonate oxygen atoms O1 and O3 to form a chain along the *c*-axis. The chains are then connected together by the bridging phosphonate oxygen O2 to form a 2D layered structure with alternating 4- and 8-membered apertures. The structure of **2** consists Yb(PMIDA) chelating units, which are connected by alternating bridging carboxylate and phosphonate groups along the [010] direction forming chains with a corrugated pattern. The third phosphonate oxygen bridges the chains together along the [001] direction to build the two-dimensional layer with 4- and 6-membered apertures in the *bc*-plane. Under excitation of 330 nm, compound **2** shows a broad emission band at λ_max_ = 460 nm. This emission is essentially in the blue luminescent region, which corresponds to ligand centered fluorescence.

## Introduction

The complexing ability of phosphonic acids have been extensively exploited in the design and synthesis of metal-organophosphonate-type metal organic frameworks (MOFs) with the formation of new and interesting compounds (Clearfield, 1988; Gagnon et al., 2012). The great interest in metal phosphonates is not only for their many unusual structural features, but also for their potential applications in different fields including adsorption, separation, gas storage, catalysis, photoluminescence, and drug delivery (Zhang and Clearfield, 1997; Sharma and Clearfield, 2000; Lukes et al., 2001; Bazaga-García et al., 2014; Liu et al., 2014). Recently, we have prepared and structurally characterized a molybdenum–diphosphonate coordination network, and shown that it can undergo reversible dehydration, which occurs with a structural change. The dehydrated material shows size selective adsorption of alcohols, adsorbing methanol but not ethanol (Ayi et al., 2013). Furthermore, we have reported crystal structures of two anionic 3D frameworks using 1,3,5-benzenetriphosphonic acid (BTP) with small amines as counter cations, Zn_2.5_(H)_0.4–0.5_(C_6_H_3_O_9_P_3_)(H_2_O)_1.9–2_(NH_4_)_0.5–0.6_(ZBP-NH_4)_, and Zn_2.5_(H)_0.75_(C_6_H_3_O_9_P_3_)(H_2_O)_2_(CH_3_NH_3_)_0.25_ (ZBP-CH_3_NH_3_), resulting from the hydrothermal synthesis using Zn(II) cations and BTP (Kinnibrugh et al., 2013). The compounds were found to exhibit a reversible dehydration process with two phase transitions resulting in a loss of volume. Research has shown that poorly crystalline metal phosphonates are common and a combination of phosphonic acids with additional carboxylic functional groups gives variety of interesting crystalline compounds (Poojary et al., 1994). The construction of multi-dimensional coordination polymers with carboxylate- and/ or phosphonate-based organic linkers has been the focus of many research groups (Galdecka et al., 2000; Paz et al., 2005; Ananias et al., 2006; Soares-Santos et al., 2006; Bao et al., 2007; Cunha-Silva et al., 2007; Girginova et al., 2007; Paz and Klinowski, 2007, 2008; Tang et al., 2007; Chelebaeva et al., 2008; Ferreira et al., 2008; Rodrigues et al., 2008; Shi et al., 2008). The use of highly flexible and/or aromatic-based organic moieties in conjunction with two or more chelating phosphonate groups has enabled the isolation of a number of structures in recent times (Serpaggi and Ferey, 1998; Evans et al., 2001; Groves et al., 2005, 2006; Ying and Mao, 2006). In the last two decades, we and others have investigated the use of a versatile chelating organic ligand: *N*-(phosphonomethyl) iminodiacetic acid (H_4_PMIDA) in the synthesis of metal carboxylate-phosphonate hybrid compounds (Zhang et al., 1996, 1998; Gutschke et al., 1999; Mao and Clearfield, 2002; Almeida Paz et al., 2004; Shi et al., 2005, 2006; Tang et al., 2006a). Using H_4_PMIDA, as a complexing agent in the presence of phosphoric acid, a mixed phosphate phosphonate layered zirconium compound was obtained by our group (Zhang et al., 1996). A linear chain compound was isolated when the reaction was carried out in the absence of phosphoric acid (Zhang et al., 1998). In both cases, the iminodiacetic moieties are only involved in hydrogen bonding, and are available for further metal complexing. An antiferromagnet K_2_Co(PMIDA)}·xH_2_O, whose crystal structure features a hexameric ring in the chair conformation was isolated by Wood and co-workers through the interaction of the salt of cobalt with H_4_PMIDA as ligand (Gutschke et al., 1999). Also with H_4_PMIDA ligand, we reported two divalent metal carboxylate-phosphonate hybrid compounds of composition [Co_2_(PMIDA)(H_2_O)_5_]·H_2_O and [Zn_2_(PMIDA)(CH_3_CO_2_H)]·2H_2_O. The structure of cobalt compound contains double layers of Co(II)carboxylate interconnected by layers of Co(II)phosphonate, while the crystal structure of zinc compound features a zinc carboxylate-phosphonate hybrid layer along the [202] plane (Mao and Clearfield, 2002). Mao and co-workers isolated isostructural lanthanide carboxyphosphonates Ln(HPMIDA)(H_2_O)_2_•3H_2_O (Ln = Gd, Tb, Dy, Y, Er, Yb, Lu), based on H_4_PMIDA anion, which exhibit a three-dimensional (3D) open-framework structures with helical tunnels (Tang et al., 2006a). Several research groups have constructed multi-dimensional frameworks by using [V_2_O_2_(PMIDA)_2_]^4−^ anionic unit, as well as a one-dimensional coordination polymer containing H_4_PMIDA residues and Fe^2+^ centers (Almeida Paz et al., 2004; Shi et al., 2005, 2006). In continuation of our investigation of the flexible coordinating properties of H_4_PMIDA, as a ligand, we extended our research to the lanthanides system. The design and syntheses of porous lanthanide phosphonates are attractive in developing new materials with multifunctions (Song and Mao, 2005; Ying and Mao, 2006; Liu et al., 2007; Mao, 2007). The successful synthesis of the first open-framework lanthanide carboxyphosphonate Pr_4_(H_2_O)_7_(O_3_PCH_2_NC_5_H_9_COO)_4_(H_2_O), opened the way for a number of other lanthanide hybrid solids to be isolated (Massiot et al., 1997; Legendziewicz et al., 1998; Serre et al., 2004; Bauer et al., 2006; Tang et al., 2006a,b; Huang et al., 2007; Zhou et al., 2010). For example, using lanthanide chlorides and *N*-(carboxymethyl)iminodi(methylphosphonic acid)(H_5_cmp) a series of layered [Ln(H_2_cmp)(H_2_O)] materials [where Ln^3+^ = Y^3+^, La^3+^, Pr^3+^, Nd^3+^, Sm^3+^, Eu^3+^, Gd^3+^, Tb^3+^, Dy^3+^, Ho^3+^, and Er^3+^], and the mixed-lanthanide [(Gd_0.95_Eu_0.05_)(H_2_cmp)(H_2_O)] material, have been successfully isolated from hydrothermal synthesis as phase-pure micro-crystalline compounds, (Cunha-Silva et al., 2009) and found to be supramolecular polymorphs of the compound that was reported by Mao and co-workers (Massiot et al., 1997; Bauer et al., 2006; Tang et al., 2006b; Zhou et al., 2010). Using phoshonoacetic acid as a complexing agent, a linear chain aluminum(III)carboxyphosphonate with ammonium ion as counter cation has been reported (Ayi et al., 2011). The ammonium cations were generated *in-situ* from the partial decomposition of urea. By employing a similar technique with the rare earth elements, we have been able to isolate two new materials (NH_4_)[La(PMIDA)(H_2_O)]•H_2_O, **1**, and (NH_4_)[Yb(PMIDA)], **2**, exhibiting 2D structures. This paper reports the synthesis and characterization of these two lanthanide(III)carboxyphosphonates.

## Experimental

### Synthesis and chemical analysis

The two compounds (NH_4_La(PMIDA)(H_2_O)•H_2_O, **1** and (NH_4_)Yb(PMIDA), **2** were hydrothermally synthesized (autogenous pressure for 6 days) at 160°C from a mixture of lanthanum chloride heptahydrate LaCl_3_.7H_2_O (Aldrich, 98%) for **1**, ytterbium oxide Yb_2_O_3_ for **2**, HCl (Fisher Scientific), *N*-(phoshonomethyl)iminodiacetic acid, H_4_PMIDA(Aldrich, 95%), potassium acetate (Fisher Scientific), urea (EM Science), and H_2_O/dioxane in the molar ratio 1:2:1: 2: 2.2: 80/10. In a typical synthesis of **1**, LaCl_3_·7H_2_O (0.557 g, 1.5 mmol) was dispersed in 2 ml of water followed by the addition of 0.26 ml HCl and 0.84 ml 1,4-dioxane. To this mixture was added H_4_PMIDA (0.34g, 1.5 mmol), urea (0.20 g, 3.3 mmol) and potassium acetate (0.10 g, 1.0 mmol). The resulting suspension with a pH of 1 was sealed in a Teflon-lined steel autoclave and heated at 160°C for 6 days. The product, a crop of colorless plate-like crystals was filtered and washed with distilled water and dried at ambient temperature. Compound **2** was obtained similarly from the composition Yb_2_O_3_(0.28 g, 1.5 mmol); 0.26 ml HCl; 2 ml H_2_O; 0.84 ml Dioxane; H_4_PMIDA(0.34 g, 1.5 mmol); and Urea (0.20 g, 3.3 mmol). The final pH was 3.5 and 2 for compounds **1** and **2**, respectively. Initial characterization was carried out by powder X-ray diffraction (PXRD), inductively coupled plasma-atomic emission spectroscopy (ICP-AES), thermogravimetric analysis (TGA), elemental CHN analysis, and IR spectroscopy. The PXRD patterns of **1** and **2** compared with their simulated patterns from single crystal analyses are presented in Figure S1. ICP-AES gave a Ln: P ratio of 1: 1 in agreement with the formula. C_5_H_14_N_2_O_9_PLa (416.06) **1**, (based on single-crystal data): calcd. La 33.55, P 7.48, C 14.43, H 3.39, N 6.73; found La 34.54, P 7.32, C 14.21, H 3.35, N 6.49; C_5_H_10_N_2_O_7_PYb (414.16) **2**, (based on single-crystal data): calcd. Yb 41.77, P 7.48, C 14.51, H 2.43, N 6.76, found Yb 40.80, P 7.18, C 14.52, H: 2.37, N 6.63. TGA data (mass losses), **1**: 43–197°C 4.72% (DTG peak at 62°C); 231–393°C 6.89% (DTG peaks at 267, 294, and 326°C); 436–893°C 41.2% (DTG peaks at 638, 755, 794, and 845°C. **2**: 260–460°C 28.82% (DTG peak at 362°C); 860–958°C 35.9% (DTG peak at 958°C); Selected ATR-FTIR data (cm^−1^), **1**: υ (O-H and N-H involved in hydrogen bonding interactions) = 3440–3000 s, (very broad), υ (C-H in –CH_2_-) = 2981 w, υ_asym_(CO^−^_2_) = 1558 vs. υ_sym_(CO^−^_2_) = 1399 s, δ (O-H… O) = 1338 m, υ (C-O) = 1247 m, υ (C-N) 1117, υ_asym_(P-O) = 1018 s, υ_sym_(P-O) = 981 s, υ (P-C) = 787 m. **2:** υ (C-H in –CH_2_-) = 2948 w, υ_asym_(CO^−^_2_) = 1593 vs. υ_sym_(CO^−^_2_) = 1405 s, 1384 s, δ (-CH_2_-) = 1448 m, υ (C-O) = 1341 m, 1333 sh, 1257 w, 1232 m, δ (C–C–N, amines) = 1154 m, υ (C-N) = 1122 w, υ_asym_(P-O) = 1084 vs. υ_sym_(P-O) = 1020 s, 1006 m, 974 w, υ (P-C) = 786 s, 713 s.

### Instrumentation

A PXRD pattern was recorded at ambient temperature on a Bruker D8 Advance diffractometer (CuK_α_ radiation λ = 1.54056 Å) fitted with Lynx EYE detector. Data were collected using a flat plate sample holder. Intensity data were collected by the continuous counting method (step 0.03° and time 3 s) in the range 5–50° 2θ. Excel and Origin 7 were used to analyse the data. Elemental analysis (C, H, and N) was performed by Atlantic Microlab, Inc. For La/P and Yb/P ratios, samples were digested in conc HNO_3_ and Anderson Analytical determined the relative amounts by ICP-AES. Thermal analysis was carried out with a Rigaku Thermoflex 8110 unit at a heating rate of 5°C/min under nitrogen atmosphere from room temperature to 1000°C. Attenuated total reflection Fourier transform infrared (ATR-FTIR) spectra (4000–400 cm^−1^) were recorded with a Perkin-Elmer 883 spectrometer.

### Crystal structure determination

Single crystal data were collected on a Bruker-AXS Apex II CCD X-ray diffractometer (MoKα radiation, λ = 0.71073 Å) operating at 110 K. The data were reduced using SAINTPLUS, (Bruker, 2005) and an empirical absorption correction was applied using the SADABS program (Sheldrick, 2008b). The structures were solved by direct methods and refined by the full-matrix least-squares technique against F^2^ with the anisotropic displacement parameters for all non-hydrogen atoms using SHELXL-2008 (Sheldrick, 2008a). All hydrogen atoms except those for the water molecules and ammonium, were added in idealized positions and refined using a riding model with *U_iso_* = *nU_eq_* for carbon atoms connected to the relevant H-atom where *n* = 1.5 for methyl and *n* = 1.2 for other H-atoms. The hydrogen atoms for the water molecules and ammonium ions in both compounds were located from difference Fourier maps and were refined using a riding mode. Anisotropic displacement parameters were established for all non-hydrogen atoms. Selected data collection and refinement parameters are summarized in Table 1. More details on crystallographic studies as well as atom displacement parameters are given in the Supporting Information (CCDC 847459) and (CCDC 847460) for compounds **1** and **2**, respectively).

**Table 1:** **Crystal data and structure refinement for 1 and 2**.

	**1**	**2**
Formula	C_5_ H_14_ N_2_ O_9_ P La	C_5_ H_10_ N_2_ O_7_ P Yb
Formula mass	416.06	414.16
Crystal system	Monoclinic	Monoclinic
Space group	P 2_1_/c	P 2_1_/c
*a* (Å)	7.059(4)	9.181(3)
*b* (Å)	23.577(12)	8.889(3)
*c* (Å)	6.871(3) Å	12.827(4)
β (°)	94.292(6)°	101.414(3)
*V* (Å^3^)	1140.4(10)	1026.0(5)
*Z*	4	4
*ρ_c_* (Mg m^−3^)	2.423	2.681
μ (Mo-K_α_)(mm^−1^)	3.931	9.294
F(000)	808	780
Reflections collected	7029	8719
Independent reflections	2460 [R(int) = 0.1268]	2454 [R(int) = 0.0718]
GOF on F^2^	1.015	1.028
Final R indices [I > 2σ (I)]^a^	R_1_ = 0.0688, wR_2_ = 0.1361 R1 = 0.1483, wR2 = 0.1680	R_1_ = 0.0321, wR_2_ = 0.0880 R_1_ = 0.0363, wR_2_ = 0.0928
Final R indices (All data)	1.849,	1.698,
(Δρ)_max_, (Δρ)_min_(e.Å^−3^)	−2.057	−2.685

a R_*1*_ = Σ ||F_o_| − |F_c_||/Σ |F_o_|. wR_*2*_ = [Σ w(F^*2*^_o_ - F^*2*^_c_)^*2*^/Σ w(F^*2*^_o_)2]^*1/2*^.

## Results and discussion

The hydrothermal treatment of H_4_PMIDA with the lanthanides (Ln = La, **1**; Yb, **2**) in the presence of HCl-urea afforded two new lanthanide(III)carboxylate-phosphonates, namely (NH_4_)[La(PMIDA)(H_2_O)]•H_2_O, **1**, and (NH_4_)[Yb(PMIDA)], **2** incorporating ammonium ions to balance the anionic framework [Ln(PMIDA)]^−^ charge of −1. The ammonium cations are generated *in-situ* from the partial decomposition of urea and are crucial in the reaction as structure directors. The pH of the reaction mixtures significantly influences the formation of the product. The initial pH for both compounds was 1.0. Whereas a final pH in the range of 3.5–4.0 favors the formation of compound **1**, a pH of 2.0 was found to favor compound **2**. The addition of urea and potassium acetate in the reaction vessel were needed to control the pH of the reaction media and to yield crystalline samples as direct addition of ammonia solution to the synthetic mixture could not lead to the formation of the products. The two compounds exhibit two-dimensional layered structures with distinct features.

**Figure 1:**
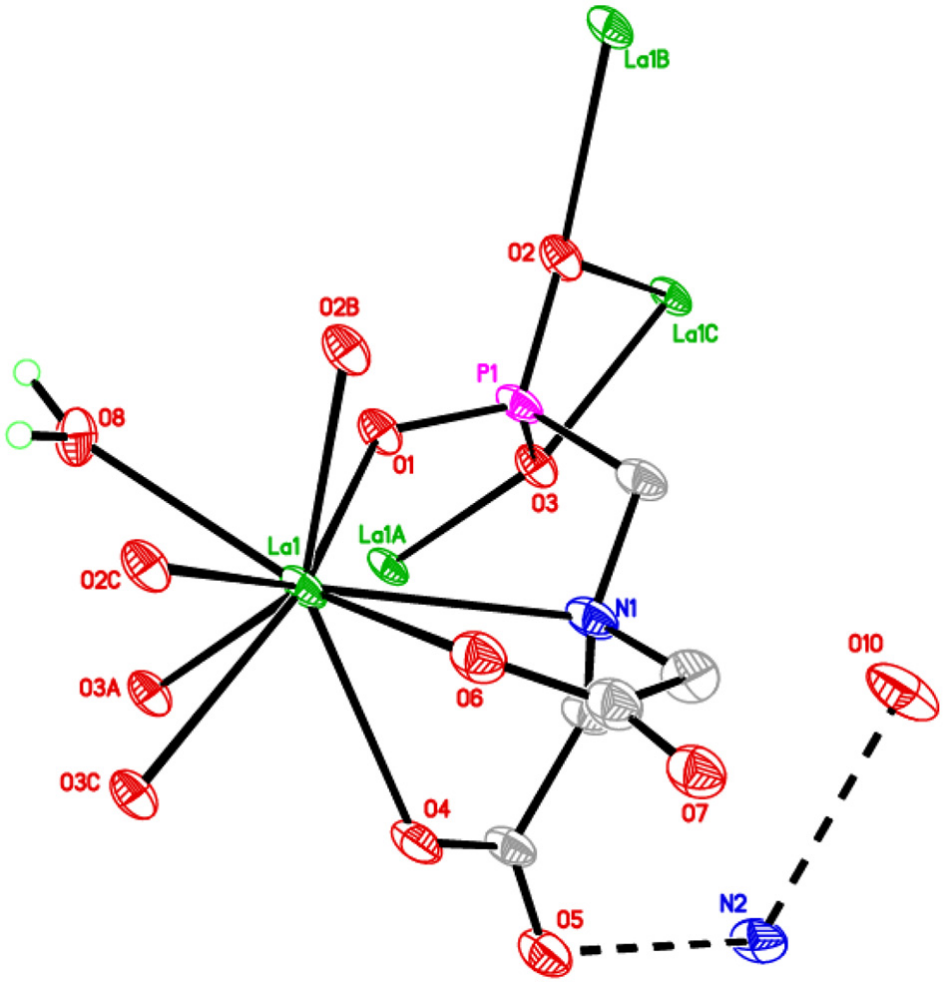
**ORTEP plot of 1 showing the labeling scheme for non-hydrogen atoms**. The thermal ellipsoids are drawn at 50% probability level. Symmetry codes for the generated atoms: (a) x, y, z−1; (b) 1−x, 2−y, 1−z; (c) x, y, 1+z.

The asymmetric unit of **1** consists of one crystallographically independent Lanthanum(III) ion, a PMIDA^4−^ anion, aqua ligand, a lattice water and an ammonium ion for charge balancing. The PMIDA^4−^ anion coordinates to the central La ion in a tetradentate fashion via one oxygen atom from each of the carboxylates (O4 and O6), one oxygen atom from the phosphonate (O1), and a nitrogen atom from the amino group (N1). This forms three five-membered chelation rings. The one independent La(III) ion is nine-coordinate and to fulfill the coordination, three other PMIDA anions coordinates to the metal center through the phosphonate oxygen atoms (O3A, O2B, O3C, and 02C) as shown in Figure 1. The geometry about the central atom is trigonal prismatic tricapped by N(1) and O(8). The interatomic distances are well defined. La-O distances are within the 2.458(9)–2.732(9) Å range [La-O_av_ = 2.568 Å] and La-N distance is 2.821(12) Å, while the C-O and P-O distances are within the 1.248(16)–1.271(16), and 1.508(9)–1.543(10) Å ranges, respectively and are indicative of complete deprotonation of both the carboxylate and phosphonate groups. The longest P-O distance belongs to the μ^2^- bridging oxygen atoms. The P-C distance is 1.791(14) Å. These distances are in good agreement with similar compounds in the literature (Legendziewicz et al., 1998; Serre et al., 2004; Song and Mao, 2005; Tang et al., 2006a; Ying and Mao, 2006; Huang et al., 2007; Liu et al., 2007; Mao, 2007).

**Figure 2:**
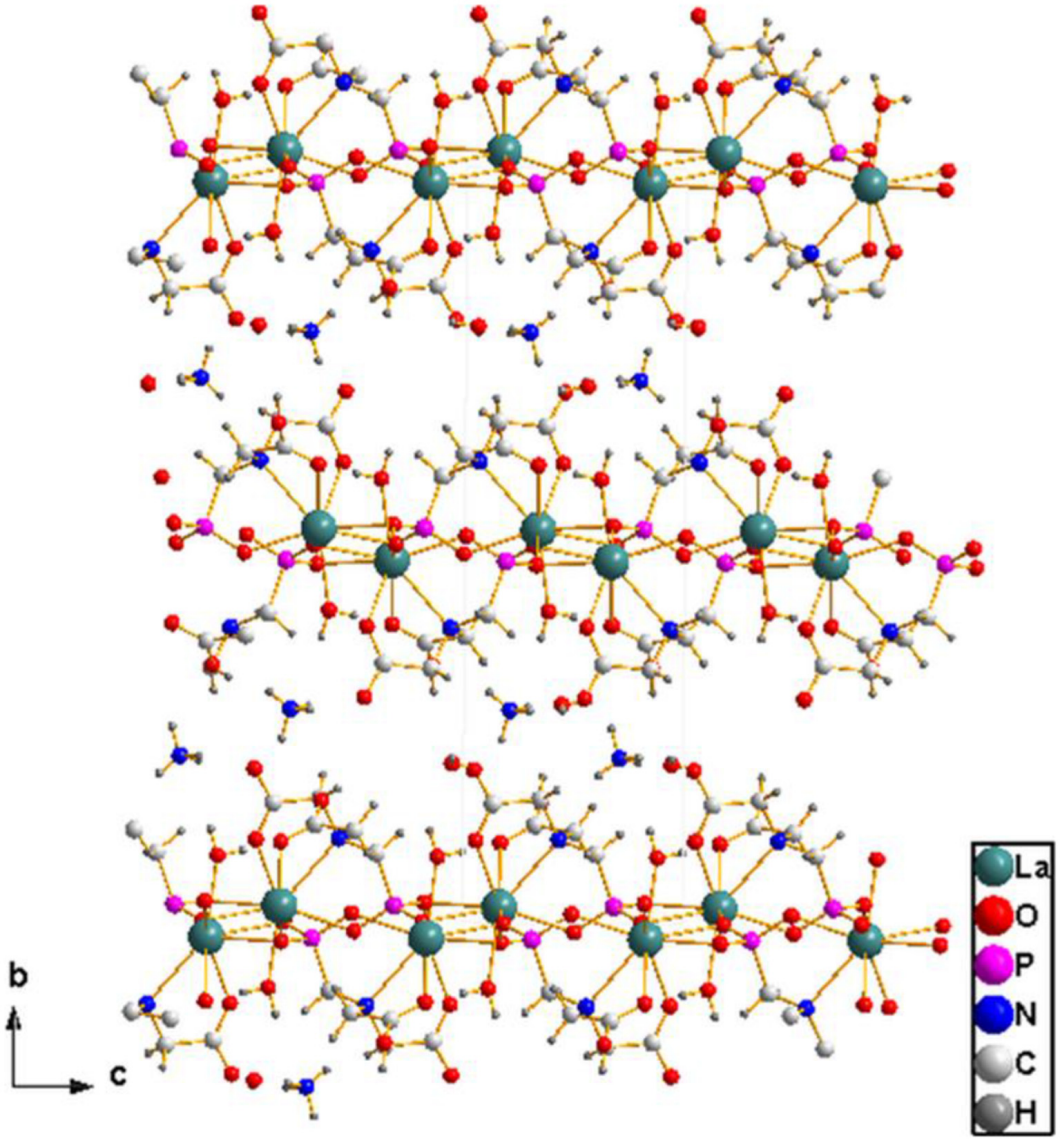
**View of compound 1 along [001] direction showing the chain structure**.

**Figure 3:**
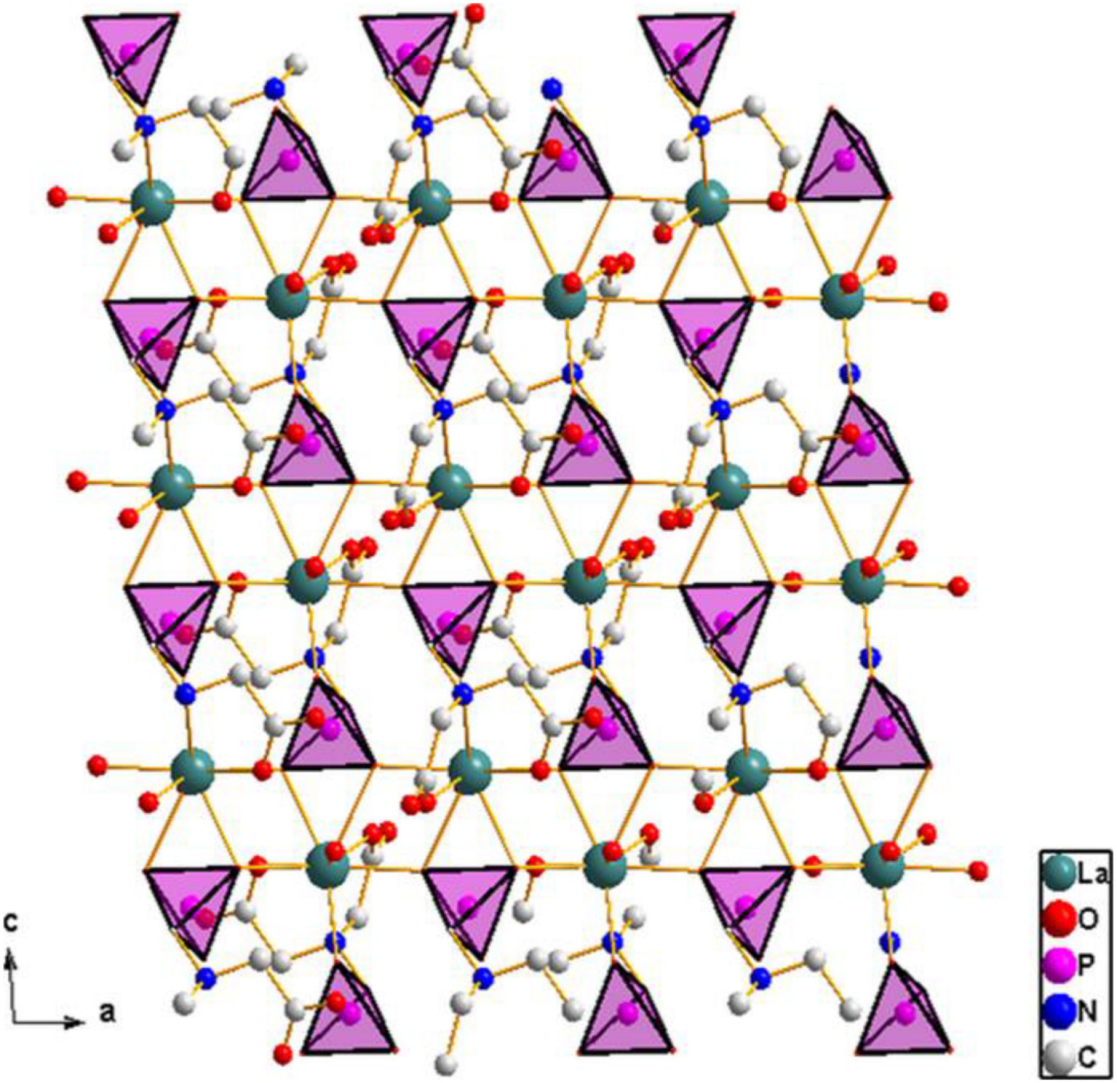
**Structure of 1 viewed along *b*-axis showing the connectivity that gives rise to 2D layer**. The lattic water and ammonium ions are ommitted for clarity.

The PMIDA^4−^ anion is a polydentate ligand, which chelates with a lanthanum(III) ion in a tetradentate fashion and bridges three other lanthanum ions using the phosphonate oxygen atoms. The phosphorus atom P(1) on the tetradentate ligand coordinating to La(1) via O1, forms multiple bonds to three equivalent lanthanum atoms [La(1A), La(1B), and La(1C)] through the remaining two oxygen atoms O2 and O3 giving rise to a [122] connectivity mode (Massiot et al., 1997; Bauer et al., 2006; Tang et al., 2006a,b; Zhou et al., 2010). While the phosphonate oxygen O2 and O3 both chelate with the La(1A) center, O3 also bridges with La(1B) to form a chain propagating along the [001] direction as shown in Figure 2. Within the chain, the La… La distances over μ_3_-O3 and O1–P1–O3 bridges are 4.227(2) and 6.871(3) Å, respectively. The chains are then connected together by the bridging phosphonate oxygen O2 to form a 2D layered structure with alternating 4- and 8-membered rings as shown in Figure 3. Each of the two carboxylate oxygen coordinates to the La(III) center in a monodentate fashion. The two non-coordinating carboxylate oxygen atoms [O5, O7] point into the interlamellar space and interact with the lattice water and ammonium ions in the interlayers via hydrogen bonds (Figure 2, Table 2). The adjacent layers separated by 12.467(6) Å, are connected together via these multiple hydrogen bonds.

**Table 2:** **Hydrogen bonds for compounds 1 and 2**.

**D-H… A**	**(D-H-A)/^**O**^**	**d(D… A)/Å**	**Symmetry operation**
**COMPOUND 1**			
N2-H2-O7	152.54	2.820(16)	1 + x, y, z
N2-H3-O5	163.65	2.825(15)	x, y, z
N2-H4-O10	168.50	2.962(14)	x, y, z
O10-H10A-O4	114.58	2.919(16)	x, y, 1 + z
O8-H8A-O6	122.54	2.656(14)	1 − x, 2 − y, 2 − z
**COMPOUND 2**			
N2-H2C-O7	167.14	2.794(5)	−1 + x, y, z
N2-H2D-O6	150.82	3.032(4)	1 − x, ½ + y, ½ − z
N2-H2E-O5	120.46	3.069(5)	1 − x, −½ + y, ½ − z
N2-H2F-O3	170.86	2.855(6)	1 − x, 1 − y, 1 − z

**Figure 4:**
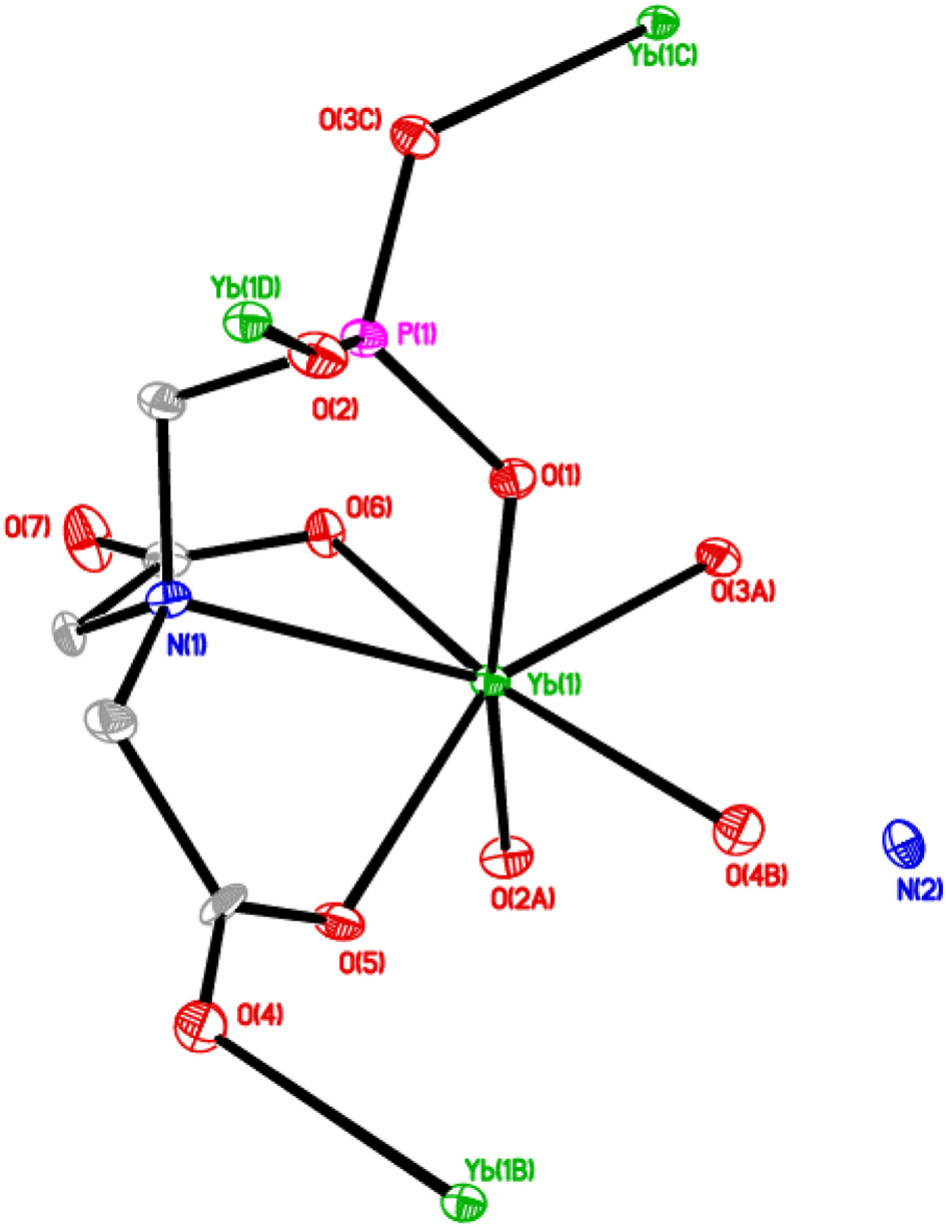
**ORTEP plot of 2 showing the labeling scheme**. The thermal ellipsoids are drawn at the 50% probability level. Symmetry codes for the generated atoms: (a) x, 0.5−y, −0.5+z; (b) 1−x, −y, 1−z; (c) 1−x, 1−y, 1−z; (d) x, 0.5−y, 0.5−z.

**Figure 5:**
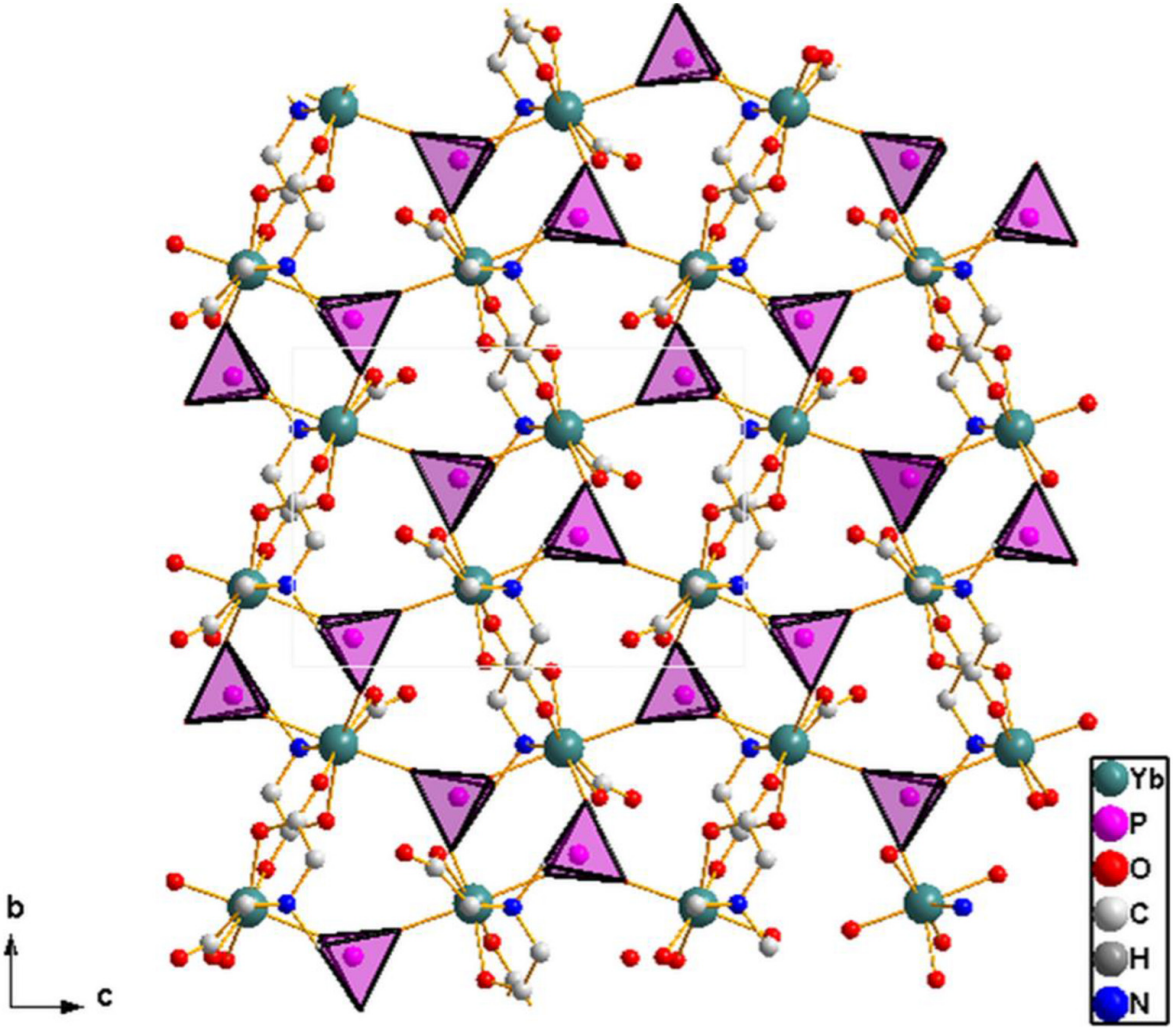
**Structure of 2 viewed along [100] direction showing the connectivity that gives the 2D layer**. Note the phosphonate group [111] connectivity mode. Ammonium ions are ommitted for clarity.

The asymmetric unit of **2** consists of one crystallographically independent ytterbium(III) ion, a PMIDA^4−^ anion, and an ammonium ion for charge balance (Figure 4). The Yb(III) ion is coordinated by a polydentate PMIDA anion in a tetradentate fashion via one oxygen atom from each of the carboxylates (O5 and O6), one oxygen atom from the phosphonate (O1), and a nitrogen atom from the amino group (N1). This forms three five-membered chelation rings similar to the lanthanum compound, **1**. Three other PMIDA anions coordinating through two phosphonate oxygen atoms (02A and 03A) and one carboxylate oxygen atom (04B) completes the coordination number of seven around the central Yb(III) ion. Interestingly, the two carboxylate groups in PMIDA^4−^ adopt different coordination modes. One carboxylate group is only coordinated to Yb atom by O6 in a monodentate fashion, while the other is bidentate bridging through O4 and O5. The geometry about the central metal ion is a capped trigonal prism YbO_6_N. The Yb-O distances are in the range 2.174(3)–2.318(3) Å, while the Yb-N distance is 2.576(5) Å and are comparable with those reported for similar compounds in the literature (Zabicky, 1970; Burwell and Thompson, 1991a,b; Tang et al., 2006a). The Yb(PMIDA) chelating units are connected by alternating bridging carboxylate and phosphonate groups along the [010] direction, forming chains with a corrugated pattern. The phosphonate oxygen O2, bridges the chains together along the [001] direction to build the layered material as shown in Figure 5.

The PMIDA^4−^ anion is a heptadentate ligand, coordinating through three phosphonate oxygen atoms (O1, O2, O3), three carboxylate oxygen atoms (O4, O5, and O6) and the amino nitrogen (N1). The phosphonate group is tridentate and bridges with three equivalent Yb(PMIDA) chelating units, that is the three oxygen atoms of the PO_3_ group are bonded to different ytterbium atoms, such that each is directly connected to only one ytterbium atom in a [111] connectivity (Massiot et al., 1997; Bauer et al., 2006; Tang et al., 2006a,b; Zhou et al., 2010). Both the Yb(III) and phosphonate group are 3-connectors in terms of topology giving the 2D layer a vertex symbol of 6 (Ayi et al., 2013), with two types of four-coordinate nodes. This kind of topology has also been observed in similar lanthanide(III) complexes, but with two types of three-coordinate nodes (Massiot et al., 1997; Bauer et al., 2006; Tang et al., 2006a,b; Zhou et al., 2010). The non-coordinating carboxylate oxygen O(7) points into the interlamellar space and interacts with the ammonium ions through hydrogen bonding (Table 2). The interlayer distance is 9.181(3) Å. Hydrogen bonding between the ammonium ions and the layers hold the layers together.

It is interesting to note that whereas the two reactions took place in aqueous solution, compound **1** has coordinated and lattice water, while compound **2** is an anhydrous complex. There is a change in coordination number from 9 in compound **1**–7 in compound **2** attributed to the decrease in the size in going from La^3+^ (103pm) to Yb^3+^ (87pm) ions (Shannon, 1976; Ma et al., 2007) Similar hydrothermal reactions of H_4_PMIDA with lanthanide(III) salts reported by Mao and coworkers gave lanthanide(III) carboxylate-phosphonates, which was formulated as Ln(HPMIDA)(H_2_O)_2_·H_2_O (Ln) Gd, **1**; Tb, **2**; Dy, **3**; Y, **4**; Er, **5**; Yb, **6**; Lu, **7**) (Tang et al., 2006a). Their structures feature a three-dimensional network with helical tunnels. In this present investigation, compound **1** is formulated as (NH_4_)[La(PMIDA)(H_2_O)]•H_2_O and compound **2** has the formula (NH_4_)[Yb(PMIDA], both featuring a two-dimensional structure with extensive hydrogen bonding involving the ammonium ion. In the previously reported compounds, the phosphonate group is singly protonated, whereas the compounds under present study shows complete deprotonation of the acidic oxygen atoms. The complete deprotonation is made possible by the addition of urea into the synthetic media, which resulted in the incorporation of ammonium ion in the present structure.

The infrared spectra of the compounds are particularly informative, providing supporting evidence for the structural differences existing between compounds **1** and **2**. The free ligand shows absorption peaks in the spectral range between 1731 and 1216 cm^−1^ arising from stretching and bending vibrational modes associated with C = O, C-O, and C-H bonds. While the band at 1731 cm^−1^ is assigned to υ_asym_(C = O), the one at 1473 cm^−1^ is due to υ_sym_(C = O). The bands at 1336, 1265, 1244, and 1216 cm^−1^ are attributed to υ_s_(C-O) whereas those at 1442 and 1423 cm^−1^ are due to δ (C-H in –CH_2_-). In the infrared spectrum of **1**, the broad band in the spectral region 3500–3360 cm^−1^ (peaking at ca. 3436 cm^−1^) is attributed to the υ (O-H) of water molecules involved in hydrogen bonds. This feature is absent in compound **2**. The broad absorption bands observed at ca. 3207 cm^−1^ (**1**) and at ca. 3180 cm^−1^ (**2**) are attributed to the υ_s_(N-H) vibrations involved in hydrogen bonding. The stretching mode of–CH_2_ groups is markedly visible in the spectra, giving rise to peak around 2981 cm^−1^ in **1** and 2948 cm^−1^ in **2**. The strong peaks observed at 1558 and 1399 cm^−1^ in **1** (1593, and 1384 cm^−1^ in **2**) are due to the asymmetric and symmetric stretching mode of the CO^−^_2_ bonds of the carboxyl group. This is lower than the 1731 cm^−1^ absorption peak seen in that of the free ligand. This down field shift in the absorption frequency is a clear indication that the carboxylate groups are involved in coordinating to the metal center (Zabicky, 1970; Nakamato, 1978; Deacon and Phillips, 1980; Burwell and Thompson, 1991a,b; Drumel et al., 1995; Ayi et al., 2011) in both **1** and **2**. In the infrared spectrum of **2**, the characteristic antisymmetric and symmetric stretching bands for the carboxylate ions are present with the corresponding Δ [ν_assym_(CO^−^_2_) − ν_sym_(CO^−^_2_)] values being 188 and 209 cm^−1^ indicating the presence of carboxylate groups in the anti-unidentate and bridging-η^2^-*anti*,*anti*-chelate coordination modes, respectively (Nakamato, 1978; Deacon and Phillips, 1980; Drumel et al., 1995). The vibrational modes of the phosphonate (PO_3_) units are also noticeable in the infrared spectra of compounds **1** and **2**. In **1**, the assymmetric stretching vibrational band of P-O group is observed at 1018 cm^−1^ and at 1084 cm^−1^ for **2**, while the symmetric stretching mode is at 981 cm^−1^ in **1** and at 1020 cm^−1^ in **2**. The P-C stretching modes are obsereved around 787 cm^−1^ in both compounds.

**Figure 6:**
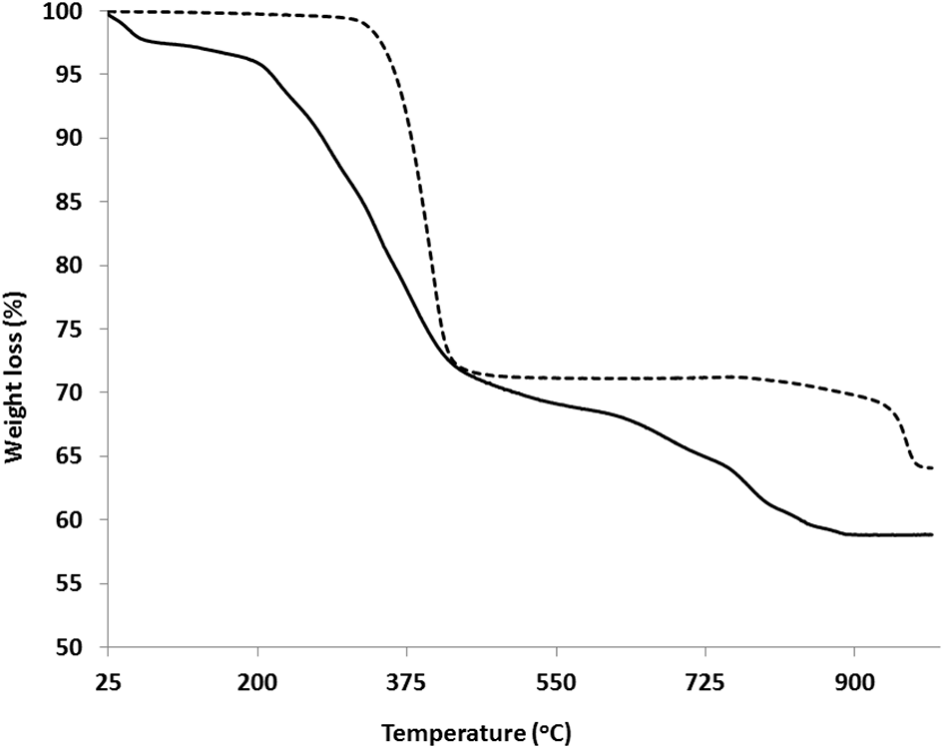
**TGA curves of compounds 1(solid) and 2 (dashed)**.

In order to investigate the thermal stability of these materials, the TGA curves of compounds **1** and **2** were measured (Figure 6). Compound **1**, releases the interlayer water molecule of crystallization in the temperature range 62–197°C. The observed weight loss of 4.62% is close to the calculated value (4.35%). In the region 202–376°C, there is a weight loss of 8.92% (calc. 8.84%) attributed to the loss of coordinated water molecule and NH_3_, as well as a loss of about 11.50% due to the presence of little impurities in the bulk sample. However, the final weight loss of 42.29% (calc. 43.78%) corresponds to the decomposition of the organic part of the material to give LaPO_4_ (JCPDF card no. 01-084-0600) as the final product. Compound **2** is thermally stable up to 300°C. The first weight loss of 28.82% (calc. 28.25%) is attributed to the loss of NH_3_, 2CO, and CO_2_. A plateau appears in the range 422–849°C, above which a final weight loss of 35.9% (calc. 35.28%) occurs, corresponding to the decomposition of the organic part to give YbPO_4_ (JCPDF card no. 01-076-1643).

**Figure 7:**
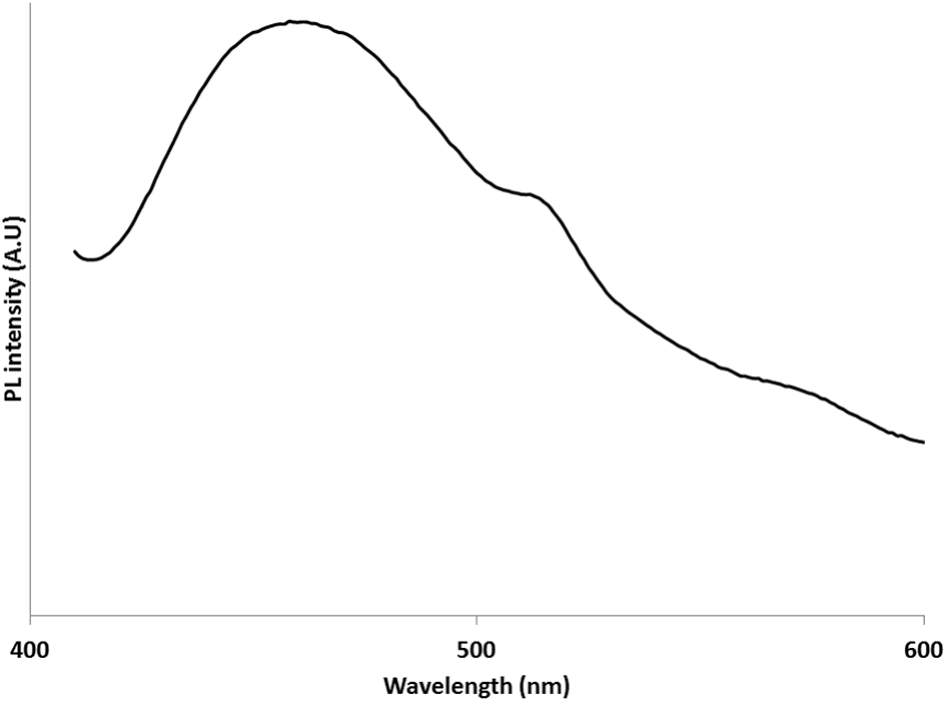
**Solid state photoluminescent studies of compound 2 at room temperature**.

The photoluminecence properties of compound **2** was investigated in the solid state at room temperature (Figure 7). Under excitation of 330 nm, the compound shows a broad emission band at λ_max_ = 460 nm, This emission is essentially in the blue luminescent region, which corresponds to ligand centered fluorescence (Tang et al., 2006a; Zhou et al., 2010). Owing to the quenching effect of the luminiscent state reported for complexes with coordinated water molecules, (Song et al., 2004; Sarkar et al., 2006; Deng et al., 2011) the solid state luminescence of **1** was not studied.

## Conclusion

We have successfully synthesized hydrothermally, two new compounds based on *N*-(phosphonomethyl)iminodiacetic acid (H_4_PMIDA), namely (NH_4_La(PMIDA)(H_2_O)•H_2_O, **1**, and (NH_4_)Yb(PMIDA), **2**. The presence of the ammonium ions serves to compensate the framework negative charge in both compounds and is crucial in the syntheses of the compounds under investigation as it stabilizes the structures through hydrogen bonding interactions. The change in the coordination number from 9 for compound **1**–7 for compound **2** clearly shows that the size of the cation plays an important role in determining the coordination number. Thus, ion with larger radius favors a higher coordination number and a larger cavity to accommodate more water molecules (Shannon, 1976; Ma et al., 2007). Solid state photoluminescent studies of compound **2** at room temperature shows broad emission in the blue luminiscent region, which is essentially attributed to ligand centered fluorescence. Efforts are under way to synthesize the complete series of the ammonium ion-templated lanthanide(III) complexes with this particular ligand with a view to elucidate their crystal structures, magnetic and luminescent properties.

### Conflict of interest statement

The authors declare that the research was conducted in the absence of any commercial or financial relationships that could be construed as a potential conflict of interest.

## Supplementary material

**Supporting Information** Available: X-ray crystallographic files in CIF format (CCDC 847459) and (CCDC 847460) for compounds **1** and **2**, respectively) as well as simulated and experimental XRD powder patterns for compounds **1** and **2**.
