# Supplementary material for: Hydrothermal synthesis and structural characterization of ammonium ion-templated lanthanide(III) carboxylate-phosphonates
Source: Front Chem. 2014 Nov 5;2:94. doi: 10.3389/fchem.2014.00094 (PMC4220733; doi:10.3389/fchem.2014.00094)
Supplement: Supplementary file 1 [file DataSheet1.DOCX]

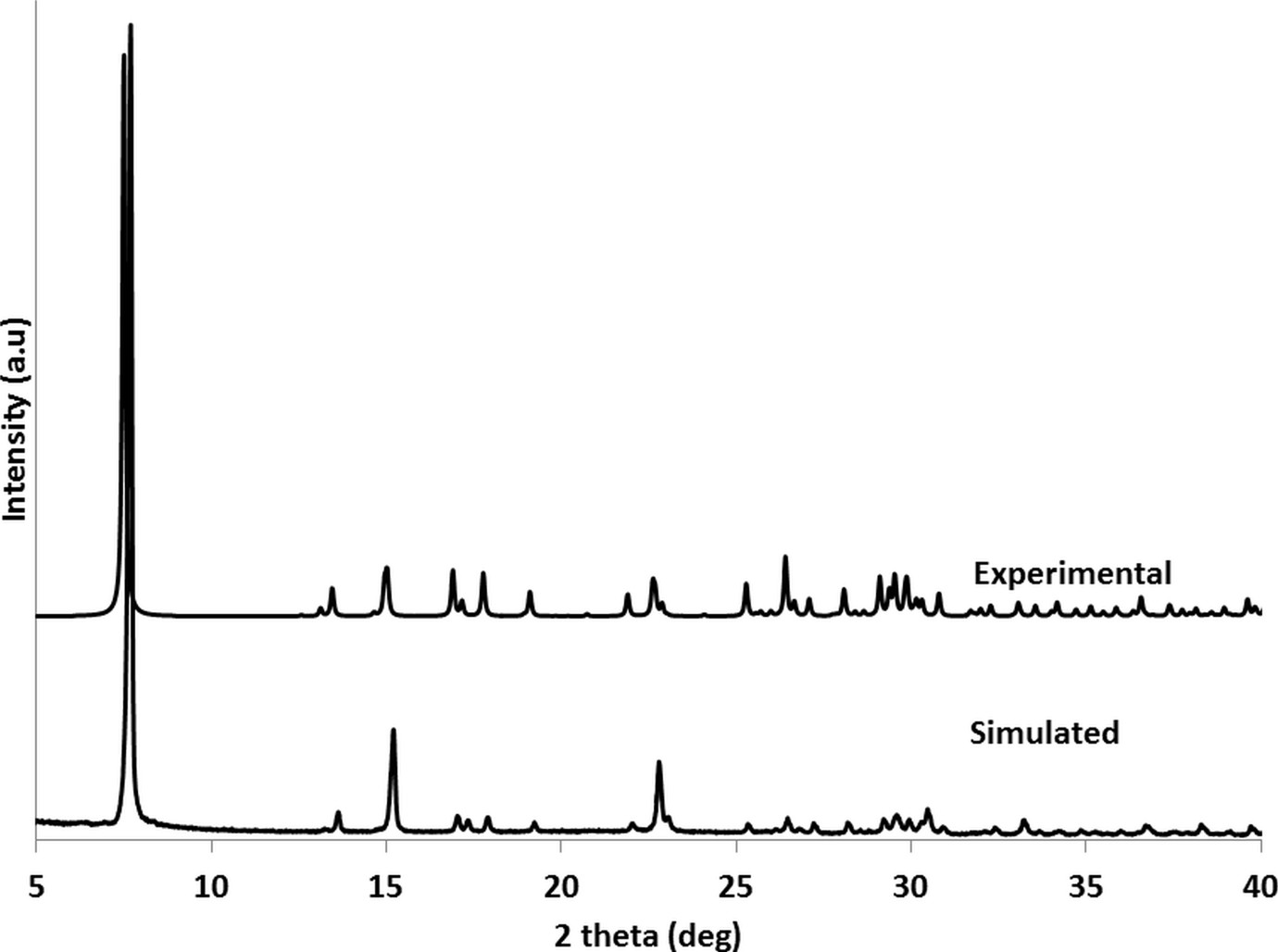


Figure S1a. Comparison of simulated and experimental PXRDs for compound **1**


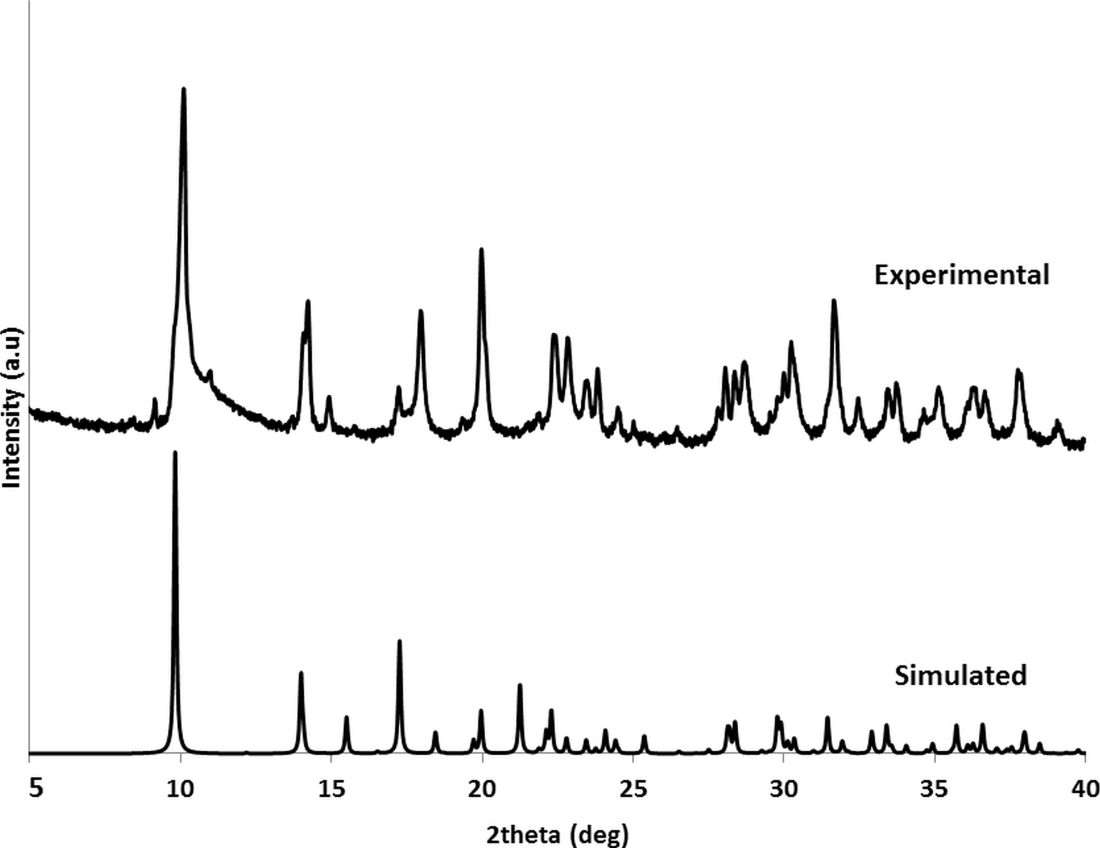


Figure S1b Comparison of simulated and experimental PXRDs for compound **2**.
